# Optical image amplification in dual-comb microscopy

**DOI:** 10.1038/s41598-020-64927-z

**Published:** 2020-05-20

**Authors:** Takahiko Mizuno, Takuya Tsuda, Eiji Hase, Yu Tokizane, Ryo Oe, Hidenori Koresawa, Hirotsugu Yamamoto, Takeo Minamikawa, Takeshi Yasui

**Affiliations:** 1 Institute of Post-LED Photonics (pLED), Tokushima University, 2-1 Minami-Josanjima, Tokushima Tokushima, 770-8506 Japan; 2 Graduate School of Technology, Industrial and Social Sciences, Tokushima University, 2-1 Minami-Josanjima, Tokushima Tokushima, 770-8506 Japan; 3 JST, ERATO, MINOSHIMA Intelligent Optical Synthesizer Project, 2-1 Minami-Josanjima, Tokushima Tokushima, 770-8506 Japan; 4 Graduate School of Advanced Technology and Science, Tokushima University, 2-1 Minami-Josanjima, Tokushima Tokushima, 770-8506 Japan; 5 Center for Optical Research and Education, Utsunomiya University, 7-1-2, Yoto, Utsunomiya Tochigi, 321-8585 Japan

**Keywords:** Confocal microscopy, Imaging and sensing, Frequency combs, Biophotonics

## Abstract

Dual-comb microscopy (DCM), based on a combination of dual-comb spectroscopy (DCS) with two-dimensional spectral encoding (2D-SE), is a promising method for scan-less confocal laser microscopy giving an amplitude and phase image contrast with the confocality. However, signal loss in a 2D-SE optical system hampers increase in image acquisition rate due to decreased signal-to-noise ratio. In this article, we demonstrated optical image amplification in DCM with an erbium-doped fiber amplifier (EDFA). Combined use of the image-encoded DCS interferogram and the EDFA benefits from not only the batch amplification of amplitude and phase images but also significant rejection of amplified spontaneous emission (ASE) background. Effectiveness of the optical-image-amplified DCM is highlighted in the single-shot quantitative nanometer-order surface topography and the real-time movie of polystyrene beads dynamics under water convection. The proposed method will be a powerful tool for real-time observation of surface topography and fast dynamic phenomena.

## Introduction

An optical frequency comb (OFC)^1–3^ is a unique optical spectrum composed of a vast number of discrete, regularly spaced optical frequency modes, and the optical frequency and phase of all OFC modes are secured to a frequency standard by active laser control of carrier-envelope-offset frequency *f*_*ceo*_ and a frequency spacing or repetition frequency *f*_*rep*_. Dual-comb spectroscopy (DCS)^4–7^ has appeared as a new mode to make full use of OFC as an optical frequency ruler for broadband high-precision spectroscopy. Use of two OFCs with slightly different frequency spacings (signal OFC, *f*_*rep1*_; local OFC, *f*_*rep2*_** =** *f*_*rep1*_** +** *∆f*_*rep*_) enables us to make a replica of the signal OFC in radio-frequency (RF) region based on a frequency scale of 1:(*f*_*rep1*_/*∆f*_*rep*_), typically 1:10^5^. The resulting mode-resolved OFC spectra of amplitude and phase have been used for broadband high-precision spectroscopy of gas^8,9^, solid^10^, and thin film^11^. Also, such DCS is available in the broad spectral range of ultraviolet^12^, visible^13^, mid-infrared^14,15^, and terahertz^6,16^, due to wavelength diversity of OFC and DCS^17^.

Recently, a new door of application has opened for DCS: spectro-imaging^18–20^ and dual-comb imaging (DCI)^21–27^. In the spectro-imaging, a combination of DCS with point-scanning imaging^18,19^ or camera-based imaging^20^ enables the hyperspectral imaging based on OFC. In DCI, OFC is regarded as an optical carrier of amplitude and phase with a vast number of discrete frequency channels in place of optical frequency ruler. Then, image pixels to be measured is spectrally encoded into OFC modes by space-to-wavelength conversion or spectral encoding (SE). Finally, image is decoded all at once from the mode-resolved spectrum of the image-encoded OFC acquired by DCS, based on one-to-one correspondence between images pixels and OFC modes. Due to the scan-less imaging capability in DCI and the simultaneous acquisition capability of amplitude and phase spectra in DCS, combination of DCI with confocal laser microscopy enables the scan-less confocal one-dimensional (1D)^21,23–25,27^ or two-dimensional (2D)^22,26^ imaging of amplitude and phase. Such dual-comb microscopy (DCM) has been effectively applied for the surface topography of a nanometer-scale step-structured sample and the non-staining imaging of standing culture fixed cells^22^.

DCM has a potential to boost the image acquisition rate up to *∆f*_*rep*_ (typically, a few kHz). While such kHz imaging rate will expand application fields of DCM into observation of fast dynamic phenomena, the largely reduced time of image acquisition leads to poor signal-to-noise ratio (SNR). Also, signal loss in a 2D-SE optical system is another reason that hampers increase in the image acquisition rate. In particular, when an objective lens with high numerical aperture (NA) is used for DCM to improve the spatial resolution, signal loss in a 2D-SE optical system is significantly increased due to the tight confocality of VIPA in the returning way. Although increase of incident optical power is a straightforward way from the viewpoint of light source, it often causes photodamage in a biological sample^28–30^. On the other hand, from the viewpoint of detector, acquisition of DCS interferogram under strong non-interferometric background light makes it difficult to use a highly sensitive photodetector. One interesting approach to enhance SNR in rapid imaging is optical amplification of image-encoded optical signal. For example, the fiber-amplifier-based image amplification was effectively applied for real-time observation of fast dynamic phenomena in serial time-encoded amplified microscopy (STEAM)^31,32^.

In this article, we adopted the optical image amplification for DCM to enhance imaging performance in rapid data acquisition or weak signal acquisition. The SNR and contrast in confocal amplitude and phase images were significantly enhanced without influence of incoherent background light of amplified spontaneous emission (ASE) by coherent amplification of image-encoded OFC interferogram in erbium-doped fiber amplifier (EDFA).

## Results

### Schematic description of optical-image-amplified DCM

**Figure 1:**
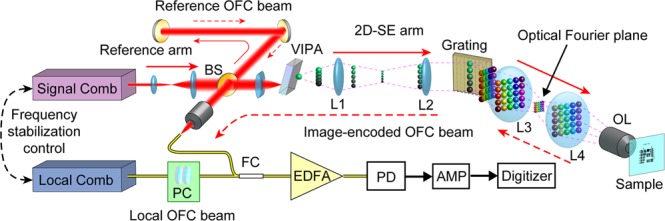
Experimental setup. BS: beam splitter, VIPA: virtually imaged phased array, L1, L2, L3, L4: lenses, OL: objective lens, FC: fiber coupler, PC: polarization controller, EDFA: erbium-doped fiber amplifier, PD: fast photodetector, AMP: Low-noise amplifier.

Figure 1 illustrates the experimental setup of the optical-image-amplified DCM, which is described in the Methods section together with details on the experimental and analytical methodology employed for the following measurements. We here give a brief description of it. An optical beam from a signal OFC (center wavelength = 1560 nm, spectral range = 1545~1575 nm, mean output power = 125 mW, *f*_*rep1*_ = 100,388,730 Hz), namely signal OFC beam, was separated into a reference arm for a *reference* OFC beam and a 2D-SE arm for an *image-encoded* OFC beam by a 50:50 beam splitter (BS), respectively^23^. The image-encoded OFC beam (mean power = 61.8 mW) passing through the BS was fed into a 2D-SE optical system^31–34^ including a virtually imaged phased array^35^ (VIPA, free spectral range = 15.1 GHz, finesse = 110) and a diffraction grating (groove density = 1200 grooves/mm, efficiency = 90%). Then it was irradiated as 2D spectrograph of signal OFC modes after passing through a pair of lenses (L3 and L4, focal length = 150 mm), and a dry-type objective lens (OL, NA = 0.95). NA of the OL used here was higher than that (= 0.25) of the OL in the previous paper^22^ to improve the spatial resolution at the expense of coupling efficiency in VIPA. While the total optical power at the sample position was 11.2 mW, the number of 2D focal spot array was 14800. Therefore, optical power per each focal spot is estimated to be 757 µW. Reflection, absorption, scattering, and/or phase change of the image-encoded OFC beam in the sample encode the image contrast onto the amplitude and phase spectra of 2D spectrograph. As the 2D spectrograph of the image-encoded OFC passed through the same optical system in the opposite direction, each wavelength component of the spectrograph was spatially overlapped with each other again as the image-encoded OFC. After the image-encoded OFC beam was combined with the reference OFC beam with a time separation of 6.2 ns by BS again, it was fed into the experimental setup of the DCS. The combined image-encoded and reference OFC beam was spatially overlapped with a local OFC beam (center wavelength = 1560 nm, spectral range = 1545~1575 nm, mean output power = 15 mW, *f*_*rep2*_ = 100,389,709 Hz, ∆*f*_*rep*_ = *f*_*rep2*_ - *f*_*rep1*_ = 979 Hz) in a single-mode fiber coupler (FC). We set an optical power ratio of the local OFC beam to the combined image-encoded and reference OFC beam to be 1:1 to obtain good visibility of the interferogram in time domain. After optical amplification by a home-made EDFA, the interferogram signal was detected by a fast photodetector (PD, bandwidth = DC to 1.2 GHz) connected with a low-noise amplifier (AMP, bandwidth = 1 kHz to 100 MHz), and was acquired by a digitizer.

A Fourier transform of the acquired interferogram signal gives the amplitude and phase spectra of the image-encoded OFC. Each data plot of the amplitude and phase spectra was spatially mapped for the confocal amplitude and phase images based on one-to-one correspondence between 2D image pixels and OFC modes^22^.

### Basic performance of EDFA for optical image amplification

**Figure 2:**
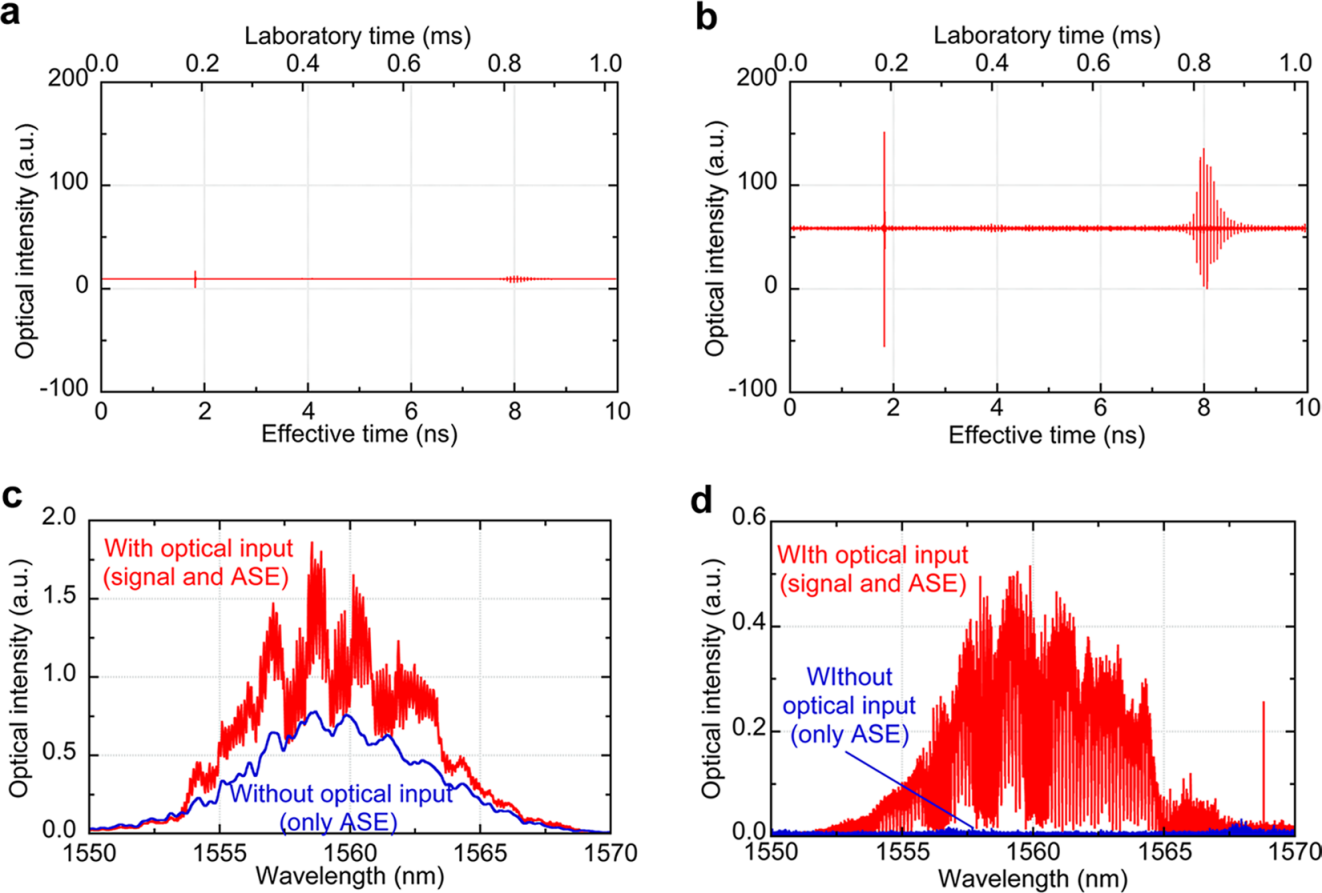
Basic performance of EDFA. Temporal waveform of interferogram (**a**) without and (**b**) with optical image amplification. (**c**) Comparison of optical spectra of EDFA output with and without the optical input of image-encoded OFC measured by an optical spectrum analyzer. (**d**) Comparison of optical spectra of EDFA output with and without the optical input of image-encoded OFC Measured by DCS.

We first investigated the time-domain performance of EDFA by measuring an interferogram between the combined image-encoded and reference OFC and the local one. A plane gold mirror was used for a sample. Figure 2a,b compare temporal waveforms of interferogram without and with optical image amplification (time window size = 1/*f*_*rep1*_ = 9.96 ns, number of signal accumulation = 1,000). In this experiment, while optical power of the signal comb with and without the EDFA was respectively 260 µW and 20 µW at the detector, that of the local comb with and without the EDFA was respectively 286 µW and 22 µW at the detector. We confirm that the photodetector has a linearity until an optical power around 500 µW. While the signal was linearly scaled with the optical power at the sample, SNR was limited by detector noise rather than shot-noise. Two interferograms appeared at 1.8 ns and 8 ns in the temporal waveform. The first interferogram, corresponding to the reference OFC, was a single burst caused by a single reflection on a mirror surface in the reference arm. The second interferogram with multiple bursts, corresponding to the image-encoded OFC, resulted from multiple reflections in the VIPA in the 2D-SE arm, and the time separation between multiple bursts was equal to an inverse of FSR. From comparison between Fig. 2a,b, amplitude of both interferograms was amplified by 13. On the other hand, when SNR of interferogram is defined as a ratio of a peak-to-peak of multiple bursts in the image-encoded interferogram to a standard deviation of noise region, SNR in the interferogram of the image-encoded OFC beam was 231.5 and 348.5 without and with the optical amplification, respectively, indicating the enhanced SNR.

When the EDFA is used with high gain, ASE is often generated in addition to the amplified input light. Such ASE might be an incoherent background light in the optical image amplification of DCM, leading to degradation of image quality. To evaluate this influence, we evaluate the  wavelength-domain performance of EDFA. A 1951 USAF resolution test chart with a negative pattern (Edmond Optics, Barrington, NJ, USA, #38–256, spatial frequency: 1.00 lp/mm ~ 228 lp/mm) was used for a sample. Figure 2c compares optical spectra of EDFA output with and without the optical input of the image-encoded OFC, measured by an optical spectrum analyzer (OSA, Yokogawa, AQ6370D-12-D/FC/RFC, wavelength = 600~1700 nm, resolution = 0.02 nm). Fine structure of red plot in Fig. 2c reflects the 2D image information of the test chart although the spectral resolution in OSA is insufficient to resolve the detailed spectral features of the image-encoded OFC. On the other hand, blue plot of Fig. 2c is corresponding to the optical spectrum of ASE. From comparison between them, the optically-amplified image-encoded OFC was spectrally overlapped with ASE background in the incoherent measurement of optical spectrum by OSA. However, such ASE background can be significantly rejected by interferometry-based coherent measurement in DCS because ASE is incoherent and is not interfered with the image-encoded OFC, reference OFC, or local OFC. Red plot and blue pot of Fig. 2d shows the optical spectra of the interferogram with and without the optical input of the image-encoded OFC, measured by the DCS. The swell of the ASE background (see blue plot in Fig. 2c) was effectively suppressed, and only fine structure of the optical spectrum reflecting the 2D image information was obtained with high contrast. When a ratio of a spectral peak of red plot to blue plot is defined as signal-to-ASE ratio, signal-to-ASE ratio was 2.39 in Fig. 2c and 16.0 in Fig. 2d, respectively. Such ASE rejection capability enables the optical image amplification in DCM without distortion or decease of the image contrast.

### Imaging performance of optically-image-amplified DCM

**Figure 3:**
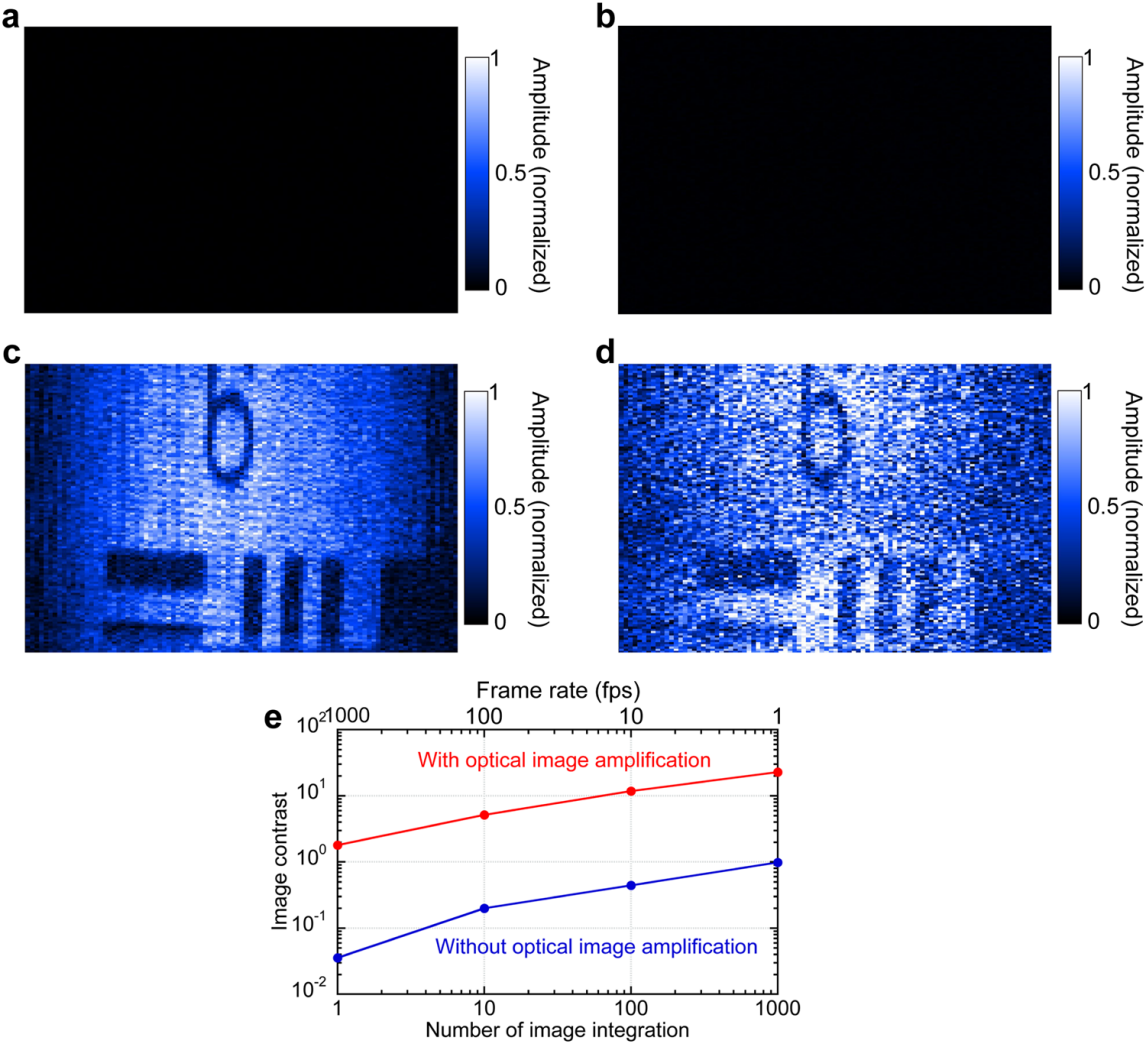
Confocal amplitude image of test chart: (**a**) without optical amplification (number of image integration = 10, corresponding to an image acquisition time of 10.2 ms or a frame rate of 97.9 fps), (**b**) without optical amplification (number of image integration = 1, corresponding to an image acquisition time of 1.02 ms or a frame rate of 979 fps), (**c**) with optical amplification (number of image integration = 10, corresponding to an image acquisition time of 10.2 ms or a frame rate of 97.9 fps), (**d**) with optical amplification (number of image integration = 1, corresponding to an image acquisition time of 1.02 ms or a frame rate of 979 fps). (**e**) Amplitude image contrast with respect to number of image accumulation.

To evaluate the effectiveness of the optical image amplification in DCM, we acquired the confocal amplitude image of the test chart with and without the optical image amplification. Figure 3a,b show confocal amplitude images (image size = 160 µm by 55 µm, image pixel = 100 pixels by 148 pixels) without the optical image amplification when the number of image accumulation was set to 10 and 1, corresponding to an image acquisition time of 10.2 ms and 1.02 ms or a frame rate of 97.9 fps and 979 fps, respectively. Since these images were obtained by removing the EDFA, we do not need to consider the effect of absorption by the erbium-doped fiber of the EDFA. In these images, no image contrast was obtained due to too weak image-encoded OFC. However, when the optical image amplification was adopted for DCM, the image contrast was significantly enhanced in the same acquisition condition as shown in Fig. 3c,d. Even in the single-shot confocal amplitude imaging of Fig. 3d, the image of the test chart was visualized moderately by the optical image amplification. Figure 3e shows the amplitude image contrast of the test chart pattern with respect to number of image accumulation. When the image contrast was defined as a ratio of a mean of amplitude difference between bright and dark regions to a standard deviation of amplitude in the bright region, the obvious enhancement of image contrast was quantitatively confirmed: 50.3 at 979 fps, 25.8 at 97.9 fps, 26.6 at 9.79 fps, and 23.2 at 0.979 fps. In this way, comparison among them clearly indicates the effectiveness of the optical image amplification in the confocal amplitude imaging of DCM.

**Figure 4:**
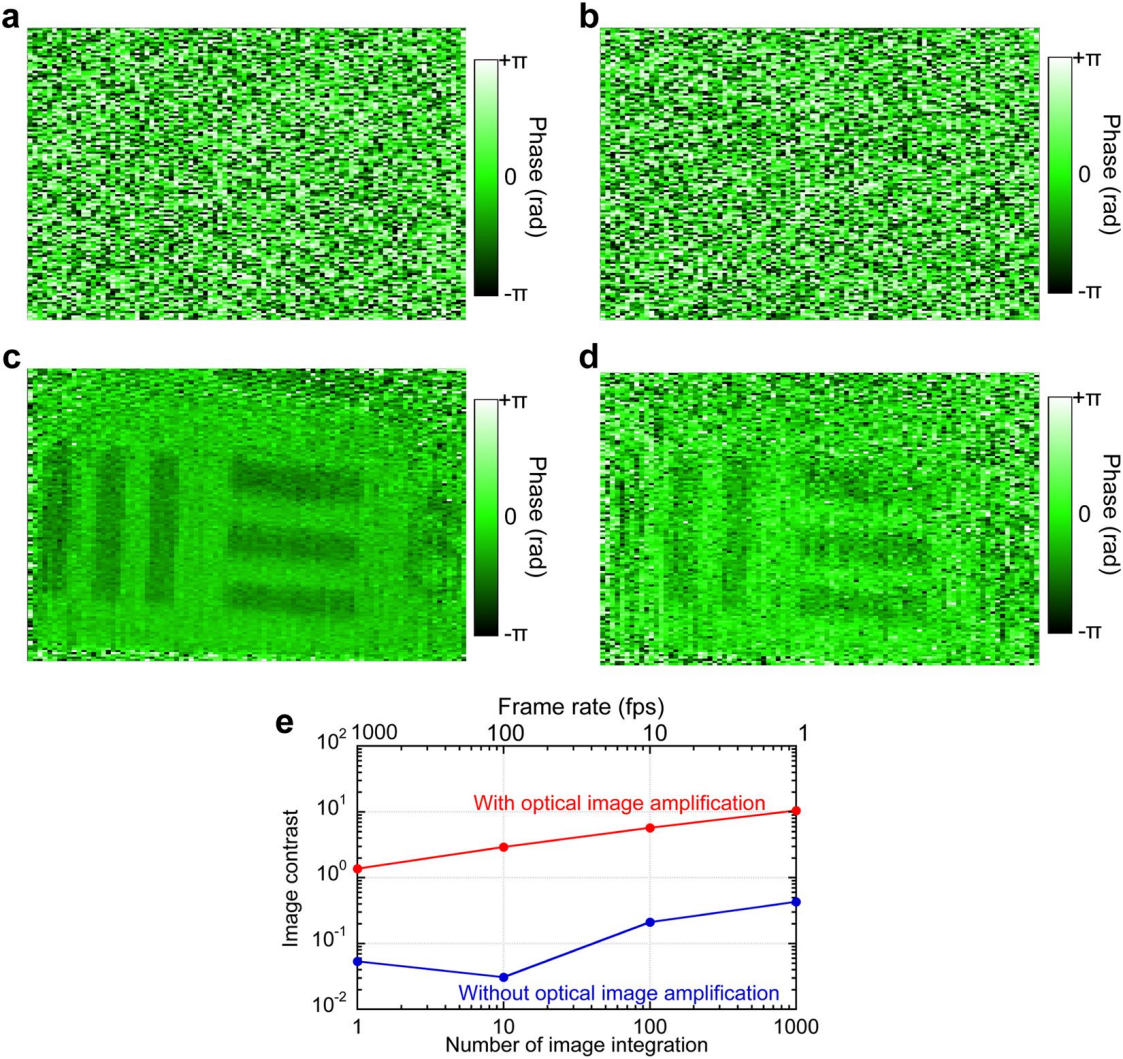
Confocal phase image of test chart: (**a**) without optical amplification (number of image integration = 10, corresponding to an image acquisition time of 10.2 ms or a frame rate of 97.9 fps), (**b**) without optical amplification (number of image integration = 1, corresponding to an image acquisition time of 1.02 ms or a frame rate of 979 fps), (**c**) with optical amplification (number of image integration = 10, corresponding to an image acquisition time of 10.2 ms or a frame rate of 97.9 fps), (**d**) with optical amplification (number of image integration = 1, corresponding to an image acquisition time of 1.02 ms or a frame rate of 979 fps). (**e**) Phase image contrast with respect to number of image accumulation.

We next evaluate the effectiveness of the optical image amplification in confocal phase imaging of DCM. Since the test chart has surface unevenness corresponding to presence or absence of reflective film, we made a thin silver coating on the test chart and used it for a sample of confocal phase imaging for a reflective sample of surface unevenness with constant reflectivity. Figure 4 shows comparison of confocal phase images with and without the optical image amplification: **4a** 10.2 ms or 97.9 fps and **4b** 1.02 ms or 979 fps without the optical image amplification, **4c** 10.2 ms or 97.9 fps and **4d** 1.02 ms or 979 fps with the optical image amplification. From comparison among them, the effectiveness of the optical image amplification was confirmed again in the confocal phase imaging. It is important to note that the single-shot confocal phase imaging is also available with the help of the optical image amplification. Figure 4e compares the phase image contrast with respect to number of image accumulation. The enhancement of phase image contrast was 25.8 at 979 fps, 95.4 at 97.9 fps, 27.2 at 9.79 fps, and 24.3 at 0.979 fps, respectively, which is similar to that of amplitude image contrast in Fig. 3e.

**Figure 5:**
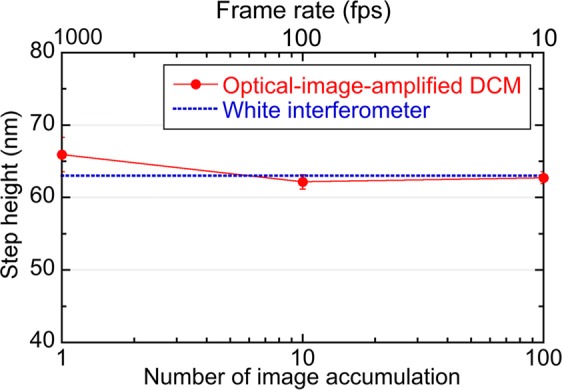
Height of the step profile with respect to the number of image accumulation.

To evaluate the quantitativeness of the rapid confocal phase imaging, we calculated surface unevenness *H(x, y)* of the silver-thin-film-coated test chart by

1 $$H(x,y)=\frac{1}{2}\frac{\phi (x,y)}{2\pi }\lambda =\frac{\lambda }{4\pi }\phi (x,y),$$

where λ is a typical wavelength of OFC modes (= 1550 nm), and *ϕ(x, y)* is the phase image. We extracted cross-sectional profile of the surface unevenness from confocal phase images with different numbers of image accumulation; then, we determined height of the step profile with respect to number of image accumulation as shown by red plots of Fig. 5. For comparison, we measured the same sample by a white-interferometer-based 3D optical profilometer (FILMETRICS Inc., Yokohama, Japan, Profilm 3D, reflection configuration, axial resolution = 0.05 µm), and determined the height of step profile to be 63 nm as shown by a blue dash line of Fig. 5. From comparison between them, the step height was correctly determined even in the single-shot confocal phase imaging.

### Confocal amplitude and phase movie of moving sample

**Figure 6:**
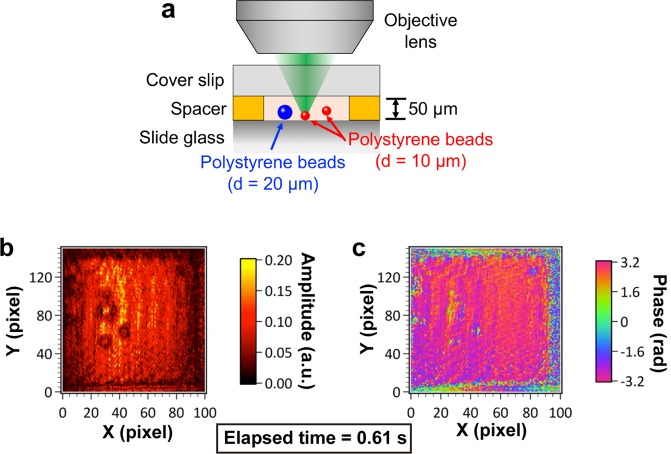
**(a)** Schematic drawing of sample. Snapshots of confocal (**b)** amplitude and (**c)** phase movie for polystyrene beads with two different diameters slowly swaying due to water convection. The corresponding movie is Video 1.

To highlight the rapid imaging capability of the optical-image-amplified DCM, we demonstrated confocal amplitude and phase imaging of a moving sample. We prepared an aggregation of polystyrene beads with two different diameters (= 10 µm and 20 µm), and put it into water contained in a glass cell for a moving sample, as shown in Fig. 6a. Figure 6b,c show snapshots of confocal amplitude and phase movie for this sample at the beginning of measurement (number of image integration = 200). The corresponding movie (frame rate = 5 fps) is shown in Video 1. These results clearly indicated polystyrene beads were slowly swaying in all directions due to water convection rather than Brownian motion. It is important to note that Fresnel reflection from the sample surface is relatively low (= 0.75%) because a refractive index of polystyrene beads (= 1.57 at 1550 nm) is similar to that of the water (= 1.32 at 1550 nm). Also, bright background in the confocal amplitude image and constant background in the confocal phase image are due to the Fresnel reflection from the rear surface of the glass cell because the focal point with confocal depth of 4.2 µm (not shown) was set just in front of the rear surface. Regardless of such low reflection, confocal amplitude and phase images clearly visualized temporal dynamics of polystyrene beads swaying near the rear surface of the glass cell benefiting from the optical image amplification. While the confocal amplitude image gives the reflective image of moving beads, the confocal phase image significantly gives the position of them within the range of confocal depth. When the phase sensitivity is defined as a standard deviation of temporal phase noise at the image pixel, it was 0.052 rad at 4.9 fps, which is corresponding to the axial position sensitivity of 6.4 nm.

## Discussion

We demonstrated effectiveness of optical image amplification in DCM for enhanced quality of confocal amplitude and phase images in the rapid acquisition rate or weak signal acquisition. We here discuss comparison between DCM and STEAM^31,32^ because 2D images pixels are superimposed on the optical spectrum by 2D spatial disperser and are amplified by fiber amplifiers in them. However, functions of fiber amplifiers are different between them: simple optical amplification in DCM and a combination of optical amplification and wavelength-to-time conversion in STEAM. Although wavelength dependence of optical amplification may change the image contrast before and after the optical amplification, it is negligible in the limited spectral bandwidth of a few tens nm. On the other hand, the residual nonlinearity of wavelength-to-time conversion may distort the STEAM image. Therefore, the optical image amplification of DCM is more robust to the image distortion than that of STEAM.

Another important difference between DCM and STEAM is in property of optical detection: coherent interferometric detection in DCM and incoherent non-interferometric detection in STEAM. While the coherent interferometric detection contributes ASE rejection capability to DCM demonstrated above, incoherent non-interferometric detection in STEAM makes it difficult to reject ASE because both signal light and ASE are acquired. Also, such the coherent interferometric detection enables us to maintain the phase image contrast after optical amplification because the phase non-linearity or phase noise in optical amplification is common between the image-encoded OFC and the local OFC due to simultaneous process of optical amplification, leading to cancellation of the phase non-linearity or phase noise in the optical amplification process. On the other hand, the non-interferometric detection in STEAM enables the background-free measurement and benefits from the high gain in the optical amplification if ASE is negligible. In the case of DCM, the background light always accompanies as non-interferometric light with the interferogram in interferometric detection, and both are optically amplified by EDFA. The amplified non-interferometric light significantly limits the dynamic range of the photodetector. As a result, the amplification ratio of EDFA was remained at 13. Such negative contribution of non-interferometric light to the photodetector dynamic range is a common problem in DCS.

We used a single photodetector to evaluate the amplified image and its quality without influence of photodetector performance in the optical amplification factor of 13. On the other hand, as a balanced photodetector is often used in DCS as well as the previous paper of DCM^22^, the comparison of image contrast between the single photodetector and the balanced photodetector is interesting. We here discuss the comparison of image contrast in the confocal amplitude image between them. For comparison, we used a balanced photodetector (Newport Corp., Irvine, California, USA, 1617-AC-FC; wavelength, 900–1700 nm; bandwidth, 40 kHz to 800 MHz). For the balanced photodetector, we set the optical amplification factor to be 34 within the range of its linear response to compensate its lower sensitivity than that in the single photodetector. Table 1 compares the image contrast in the confocal amplitude image (number of signal accumulation = 1,000) among the single photodetector without and with optical image amplification and the balanced photodetector without and with optical image amplification. Enhancement of image contrast by optical image amplification was confirmed in both detectors. Also, the enhancement of image contrast in the balanced photodetector was larger than that in the single detector although the optical amplification factor was different between them. On the other hand, when comparing the image contrast between the single photodetector with optical image amplification and the balanced photodetector without the optical image amplification, the optical image amplification was more powerful than that the balanced photodetector. The reason why the benefit of the balanced photodetector is not so clear may be that the rejection capability of the common-mode optical intensity noise by the balanced detection was somewhat lost in the lower sensitivity and inherent electric noise of the balanced photodetector under the detection of weak optical signal.

**Table 1:** Comparison of image contrast in confocal amplitude image.

	Image contrast without optical image amplification	Image contrast with optical image amplification	Enhancement of image contrast
Single photodetector	5.6	23	4.1
Balanced photodetector	1.5	19.5	13

One may consider that the spectral resolution of VIPA is insufficient for separation of each OFC mode. From free spectral range of 15.1 GHz and finesse of 110 in VIPA, the spectral resolution of VIPA is estimated to be 137 MHz, indicating that a few OFC modes are included in each spot of the image domain. However, in the frequency domain, DCS can separately acquire OFC mode of amplitude and phase. In this way, even though it is difficult to separate each mode in the image domain, one can separate each mode in the frequency domain.

We finally discuss the extension of the optical-image-amplified DCM to samples of scientific interest. While DCM benefits from scan-less imaging, full-field confocality, and the optical-phase-based image contrast, introduction of optical image amplification in DCM enables the confocal amplitude and phase imaging under weak light detection or rapid data acquisition. These characteristics will be useful for applications requiring the precise time synchronization within the image. For example, although temporal dynamics of cellular and intracellular response are interesting topics in life science, the frame rate in usual confocal laser microscopy (≈10 frame/s) is insufficient for full analysis of them. In the demonstration of Fig. 6, when regarding moving polystyrene beads in the water as living cells in the solution with similar transparency and refractive index, spatio-temporal imaging of cellular and intracellular dynamics is a potential application in life science. On the other hand, since micro-electro-mechanical-systems (MEMS) devices work with millisecond-order vibration and sub-micrometer-order displacement, analysis of vibration and displacement in MEMS devices is another potential application in industrial inspection because rapid and precise phase-imaging capability (see Fig. 5) enables surface topography of moving objects.

In summary, optical image amplification was successfully introduced in DCM for confocal amplitude and phase imaging in the condition of the rapid acquisition rate or weak signal acquisition. Optical amplification of the interferogram in EDFA significantly enhances the quality of the confocal amplitude and phase images without influence of ASE background. At single-shot acquisition of phase image, high quantitative values were confirmed in nanometer-order surface topography based on the confocal phase imaging. Furthermore, temporal dynamics of polystyrene beads in water was visualized in the confocal amplitude and phase movie. The optical-image-amplified DCM will be a powerful tool for real-time observation of surface topography and fast dynamic phenomena of cells.

## Methods

### Experimental setup

Figure 1 shows an experimental setup of the optical-image-amplified DCM system. Since the detail of DCM without the optical image amplification is described in the previous paper^22^, we here give a brief description of it. We used a pair of homemade femtosecond Er-fiber OFC lasers for a signal OFC (center wavelength = 1560 nm, spectral range = 1545~1575 nm, mean output power = 125 mW, *f*_*ceo1*_ = 21.4 MHz, *f*_*rep1*_ = 100,388,730 Hz) and a local OFC (center wavelength = 1560 nm, spectral range = 1545~1575 nm, mean output power = 15 mW, *f*_*ceo2*_ = 21.4 MHz, *f*_*rep2*_ = 100,389,709 Hz, ∆*f*_*rep*_ = *f*_*rep2*_ - *f*_*rep1*_ = 979 Hz) in DCM. While *f*_*ceo1*_ and *f*_*ceo2*_ were controlled by a current of pumping laser diodes for OFCs, *f*_*rep1*_ was controlled by a piezoelectric actuator attached to a cavity fiber of the signal OFC. *f*_*ceo1*_, *f*_*ceo2*_, and *f*_*rep1*_ were all phase-locked to a rubidium frequency standard (not shown, Stanford Research Systems, Inc., Sunnyvale, CA, USA, FS725, frequency = 10 MHz, accuracy = 5 × 10^−11^, instability = 2 × 10^−11^ at 1 s) via a laser control system. The local OFC laser had an intra-cavity electro-optical modulator for laser control^36^ and was tightly and coherently locked to the signal OFC to maintain *Δf*_*rep*_ at 979 Hz using a narrow-linewidth continuous-wave (CW) laser (Redfern Integrated Optics, Inc., Santa Clara, California, USA, PLANEX; center wavelength, 1550 nm; FWHM, < 2.0 kHz) for an intermediate laser^10,37^. This locking enabled us to coherently accumulate interferograms obtained with DCS and hence to enhance the SNR^38,39^.

The signal OFC beam was separated into a reference arm for a *reference* OFC beam and a 2D-SE arm for an *image-encoded* OFC beam by a 50:50 beam splitter (BS), respectively^23^. The reference OFC beam in the reference arm was reflected by a gold plane mirror and was combined with the image-encoded OFC beam by BS. To separate an interferogram of the reference OFC from that of the image-encoded OFC temporally, we adjusted difference of optical path length between the reference arm and the 2D-SE one. In the 2D-SE arm, the image-encoded OFC beam (mean power = 61.8 mW) was fed into a 2D-SE optical system^31–34^, composed of a virtually imaged phased array^35^ (VIPA, Light Machinery, Inc., Nepean, Ontario, Canada, OP-6721-6743-8, free spectral range = 15.1 GHz, finesse = 110), a diffraction grating (Spectrogon AB, Täby, Sweden, PC 1200 30 × 30 × 6, groove density = 1200 grooves/mm, efficiency = 90%), and lenses (L1, focal length = 200 mm; and L2, focal length = 100 mm; L3, focal length = 150 mm). The 2D-SE optical system forms 2D spectrograph of signal OFC modes at an optical Fourier plane. The 2D spectrograph was relayed and focused as 2D focal spot array of signal OFC modes onto a sample by a combination of a lens (L4, focal length = 150 mm) with a dry-type objective lens (OL, Nikon Corp., Tokyo, Japan, Plan Apo Lambda 40XC, numerical aperture = 0.95, working distance = 160~250 µm). Reflection, absorption, scattering, and/or phase change of the signal OFC beam in the sample encode the image contrast onto the amplitude and phase spectra of 2D spectrograph. As the 2D spectrograph of the image-encoded OFC passed through the same optical system in the opposite direction, each wavelength component of the spectrograph was spatially overlapped with each other again as an image-encoded OFC. Typical power of the image-encoded OFC was decreased down to a few tens to a few hundred µW mainly due to VIPA passage in the return path. After being spatially overlapped with the reference OFC beam by BS, the combined reference and image-encoded OFC beam was fed into the experimental setup of the DCS.

The combined reference/image-encoded OFC beam was spatially overlapped with the local OFC beam in a single-mode fiber coupler (FC). The split ratio of 95:5 for the combined reference/image-encoded OFC beam and the local OFC beam in FC was selected considering non-negligible signal loss in the 2D-SE optical system. A polarization controller (PC) in the local OFC path was adjusted to enhance the interferogram signal between the signal and local OFC beams via high polarization overlapping between them in the fiber. The resulting interferogram signal was amplified by a forward-pumping EDFA composed of a 4.5 m length of erbium-doped fiber (nLIGHT Inc., Vancouver, WA, USA, LIEKKI ER30-4/125, peak core absorption at 1530 nm = 30 dB/m) and a pumping laser diode (Thorlabs Inc., Newton, NJ, USA, BL976-PAG900, wavelength = 980 nm, power = 900 mW). The optically amplified interferogram was detected by a fast photodetector (PD, Thorlabs Inc., Newton, NJ, USA, DET01CFC-N, wavelength = 800~1700 nm, bandwidth = DC to 1.2 GHz) connected with a low-noise amplifier (AMP, bandwidth = 1 kHz to 100 MHz). The detected electrical signal was acquired using a digitizer (National Instruments Corp., Austin, TX, USA, NI PXIe-5122, resolution = 14 bit). The sampling clock signal was synchronized with *f*_*rep2*_. We acquired a temporal waveform of interferogram signals with a time widow of 9.96 ns (= 1/*f*_*rep1*_) and a sampling interval of 97 fs (= 1/*f*_*rep1*_−1/*f*_*rep2*_).

Two interferograms, caused by the reference OFC and the image-encoded OFC, appeared with a time separation of 6.2 ns in the temporal waveform as shown in Fig. 2a,b. We separated the temporal waveform into two parts to include one interferogram in each part. Then, we performed the Fourier transform of them, giving the amplitude and phase spectra of the reference and image-encoded OFCs. We subtracted the phase spectrum of the reference OFC from the phase spectrum of the image-encoded OFC to eliminate the influence of the initial phase in the signal OFC and/or common phase noise between dual OFCs. Then, each data plot of the amplitude and phase spectra was spatially mapped for the confocal amplitude and phase images based on one-to-one correspondence between 2D image pixels and OFC modes^22^.

## Data Availability

The data supporting the findings of this study are available from the corresponding author upon reasonable request.
